# Gonadal transcriptome sequencing reveals sexual dimorphism in expression profiling of sex-related genes in Asian arowana (*Scleropages formosus*)

**DOI:** 10.3389/fgene.2024.1381832

**Published:** 2024-04-11

**Authors:** Chenxi Zhao, Chao Bian, Xidong Mu, Xinhui Zhang, Qiong Shi

**Affiliations:** ^1^ College of Life Sciences, University of Chinese Academy of Sciences, Beijing, China; ^2^ Shenzhen Key Lab of Marine Genomics, Guangdong Provincial Key Lab of Molecular Breeding in Marine Economic Animals, BGI Academy of Marine Sciences, BGI Marine, Shenzhen, China; ^3^ Laboratory of Aquatic Genomics, College of Life Sciences and Oceanography, Shenzhen University, Shenzhen, China; ^4^ Key Laboratory of Prevention and Control for Aquatic Invasive Alien Species, Ministry of Agriculture and Rural Affairs, Guangdong Modern Recreational Fisheries Engineering Technology Center, Pearl River Fisheries Research Institute, Chinese Academy of Fishery Sciences, Guangzhou, China

**Keywords:** Asia arowana, gonad transcriptome, sex-biased, *foxl2*, *dmrt* gene family

## Abstract

Asia arowana (*Scleropages formosus*) is an ornamental fish with high economic value, while its sex determination mechanism is still poorly understood. By far, no morphological evidence or molecular marker has been developed for effective distinguishment of genders, which poses a critical challenge to our captive breeding efforts. In this study, we sequenced gonadal transcriptomes of adult Asian arowanas and revealed differential expression profiling of sex-related genes. Based on the comparative transcriptomics analysis of testes (n = 3) and ovaries (n = 3), we identified a total of 8,872 differentially expressed genes (DEGs) and 18,490 differentially expressed transposable elements (TEs) between male and female individuals. Interestingly, the expression of TEs usually has been more significantly testis-biased than related coding genes. As expected, several genes related to females (such as *foxl2* and *cyp19a1a*) are significantly transcribed in the ovary, and some genes related to male gonad development (such as *dmrt1*, *gsdf* and *amh*) are highly expressed in the testis. This sexual dimorphism is valuable for ascertaining the differential expression patterns of sex-related genes and enriching the genetic resources of this economically important species. These valuable genetic materials thereby provide instructive references for gender identification and one-to-one breeding practices so as to expand fish numbers for a rapid elevation of economic value.

## Introduction

Teleost exhibit remarkable diversity of sex determination mechanisms, including genetic sex determination (GSD; Myosho et al., 2012), environmental sex determination (ESD; Li et al., 2023), and various combinations of these two modes. In the GSD system (such as XX/XY and ZW/ZZ), the expression of sex-determining genes initiates a series of cascadic signaling pathways of sex determination and differentiation, thereby inducing the primordial gonads to develop into ovaries or testes (Li and Gui, 2018). Many master sex-determining genes or sex differentiation-related genes have been reported to play a main role in regulation of sex development in various fishes, such as *dmrt1* (doublesex and mab-3 related transcription factor 1), *sox9* (SRY-box transcription factor 9), and *foxl2* (forkhead box protein L2). Some transcription factors, involved in regulating gene expression, play a crucial role in the development of male or female differentiation (Cocquet et al., 2002; Cui et al., 2017; Hu et al., 2021).

In fish, *dmrt1* is the master sex-determining gene in half-smooth tongue sole (Cui et al., 2017), and its homologous gene *dmy* (the *dmrt1* paralogous located in Y-chromosome) was verified to be the sex-determining gene in medaka (Nanda et al., 2002; Kobayashi et al., 2004). Nearly all polypeptide chains encoded by *dmrt* genes contain a highly conserved zinc finger DNA-binding and transcription regulating motif (known as DM domain), which consists of six conserved cysteines and two histidines for binding into the minor groove of any target DNA. High expression of *dmrt1* in males activates genetic programs to promote testicular differentiation. There is only one copy of the *dmrt2* gene in mammals, but two copies (*dmrt2a* and *dmrt2b*) are commonly identified in teleost fishes. It has been reported that the *dmrt2* (a/b) genes are involved in determination of the body axis and segment differentiation of vertebrates and are also related to the gonadal sex differentiation process (Han et al., 2021). In bony fishes, *dmrt3* is usually highly expressed in the testis and nervous system, which presumably plays an important role in the differentiation and development of nerve and germ cells as well as testes (Li et al., 2008; Dong et al., 2010). In contrast, in females, high expression of *foxl2* inhibits the expression of *dmrt1* while upregulates *cyp19a1*, thereby promoting the development of ovarian tissues (Zhang et al., 2017). The transforming growth factor-β (TGF-β) signaling pathway mainly participates in mediating the formation of tissues and organs as well as reproductive development by regulating cell growth, proliferation, differentiation and other processes (Li and Gui, 2018; Pan et al., 2021). Many previous studies have proved that this signaling pathway is related to sex determination and differentiation in various fishes. Meanwhile, some members of the TGF-β superfamily have been considered as initiators or key regulators of sexual differentiation, such as *amh* (anti-Müllerian hormone), *amhr2* (anti-Müllerian hormone receptor type 2; (Kamiya et al., 2012), and *gsdf* (gonadal soma-derived factor; (Myosho et al., 2012).

Transposable elements (TEs) have been integral parts of vertebrate genomes throughout the evolution process and play important roles in promoting genetic innovation, sex determination and reproduction (Biémont and Vieira, 2006). Recent bursts of TEs and simple repeat accumulations were observed around young sex determination loci, indicating the strong association between TE amplification and the formation of sex determination regions on sex chromosomes (Chalopin et al., 2015). TEs could carry regulatory elements and modify the expression of neighboring genes, thereby participating in the evolution of germ cells and gonadal regulatory networks. The expression and transposition of TEs are critical for vertical transmission to progeny and persistence in lineages (Dechaud et al., 2019). Interestingly, TEs are particularly prone to being recruited in sexual development, since they are typically expressed in the gonads (Brunet et al., 2018). For instance, in *Oryzias latipes* approximately 1.2% and 3.5% of ovarian and testicular transcripts were reported to be attributed to TE expression, respectively (Dechaud et al., 2019).

Asian arowana (*Scleropages formosus*, also known as dragonfish) belongs to the ancient family Osteoglossidae, and it is a freshwater species with a native distribution in Southeast Asia. It has become a well-known ornamental fish for its vibrant colors and cultural significance. Mainly due to habitat destruction and overfishing, arowanas have been classified as endangered species in Appendix I by the Convention on International Trade in Endangered Species of Wild Fauna and Flora (CITES). In recent years, significant progress has been made in the genetics research and breeding techniques of arowanas (Chang, 2009; Mu et al., 2012; Shen et al., 2014; Austin et al., 2015; Bian et al., 2016; Mu et al., 2022).

Asian arowana often reaches sexual maturity at 2–4 years of age (Chang, 2009), and several previous studies proposed that the genetic sex determination system of this species may be ZW/ZZ (Shen et al., 2014; Bian et al., 2016). Because there are no obvious morphological differences during all life stages of the Asian arowana, even after sexual maturity, it is very difficult to phenotypically differentiate the sex of individuals. One recent report showed that its putative ZW system is more likely a polymorphic pattern that occurs in the 18th chromosome pair, including the accumulation of constitutive heterochromatin and 18S rDNA (Toma et al., 2023). Meanwhile, although its genomes of both sexes have been published by us (Mu et al., 2022), our in-depth comparative genomics between males and females has failed to identified any significant region with obvious genomic differences. Therefore, we performed this study to identify coding and non-coding regions that are differentially expressed between ovaries and testes of adult Asian arowanas through transcriptome sequencing, which may become differential signatures between both sexes for practical gender identification and one-to-one captive breeding.

## Materials and methods

### Sample collection

Three female and three male individuals of Asian arowana, around 5-year-old, were obtained from Pearl River Fisheries Research Institute, Chinese Academy of Fishery Sciences (Guangzhou, Guangdong, China). After dissection, the sex of these fishes was determined based on morphological inspection of the gonads. A total of six gonad tissues, including three ovaries and three testes, were frozen in liquid nitrogen immediately and stored at −80°C until use.

### RNA isolation, library construction, and transcriptome sequencing

Total RNAs were extracted from the ovary and testis tissues by using a TRIZOL Kit (Invitrogen, Carlsbad, CA, United States of America) following the manufacturer’s instructions. The extracted RNAs were then digested by DNase I to eliminate genomic DNA’s contamination. Purified RNA integrity and quality were assessed with an Agilent 2,100 Bioanalyzer System (Agilent Technologies, Santa Clara, CA, United States of America). Only those RNA samples with RIN (RNA integrity number) > 7.0 were utilized for library construction. A total of six cDNA libraries with insert sizes of 300–400 bp were generated in DNA nanoballs (DNBs) according to the manufacturer’s protocol of DNBSEQ sequencing platform, and then sequenced on a MGISEQ-2000 platform (MGI, BGI Shenzhen, China) to obtain 150-bp paired-end reads.

### Screening and functional analysis of sex-biased differentially expressed genes (DEGs)

Raw reads with low quality, adapter sequences, and/or highly unknown N bases were filtered by using SOAPnuke v1.5.6 with optimized parameters “filter -n 0.01 -L 15 -q 0.4 -G -Q 2” (Chen et al., 2017). The clean RNA reads from six samples were subsequently aligned onto the previously published female genome assembly (Mu et al., 2022) by using STAR v2.5.3 (Dobin et al., 2013), and then RSEM v1.2.8 (Li and Dewey, 2011) was applied to quantify transcription levels of genes and transcripts. The Fragments Per Kilobase of exon model per Million mapped fragments (FPKM) algorithms were used to normalize the mRNA expression levels. Based on the quantitative data, we utilized the DESeq2 R package (Love et al., 2014) to characterize DEGs between ovaries and testes. Adjusted *p*-value (false discovery rate, FDR) < 0.05 and absolute value of log_2_ (fold change) > 1.5 were assigned as the stringent threshold for significant DEGs (Stachowiak et al., 2024).

Functional annotations of the DEGs were performed via the NCBI NR database, Gene Ontology (GO) (Ashburner et al., 2000) enrichment analysis and Kyoto Encyclopedia of Genes and Genomes (KEGG) (Kanehisa and Goto, 2000) pathway enrichment analysis. In addition, we compared all genes to the AnimalTFDB2.0 (Zhang et al., 2015) database to obtain transcription factor (TF) families. We subsequently applied DIAMOND (Buchfink et al., 2015) to align the DEGs with the sequences of Asia arowana in the STRING v12.0 database (Szklarczyk et al., 2023), constructed a potential protein-protein interaction (PPI) network among potential sex-related genes, and visualized the results using Cytoscape v3.10.1 (Su et al., 2014).

### Quantification of TE expression

To analyze the expression of TEs, we applied TEtranscripts v2.2.3 (Jin et al., 2015) to estimate TE expression at a copy-level resolution by using sorted bam files from STAR. TE quantification was performed using RepeatMasker (Tarailo-Graovac and Chen, 2009) TE annotation. TE transcripts were used for a differential expression analysis between ovary and testis tissues with DESeq2 (Love et al., 2014) to generate a normalized count matrix, variance-stable count matrix, and matrix of differential gene expression. We further classified TE categories into LTRs, LINEs, SINEs, DNA TEs, and unknowns as provided in the published TE annotation of arowana (Mu et al., 2022). The numbers of differentially expressed TEs by sex within each category were reported for ovaries and testes.

To characterize the distributions of sex-biased genes and TEs across the arowana genome, we examined whether sex-biased genes clustered on the genome according to a published pipeline (https://gitlab.com/Corend/gene_clusters_pyth) (Dechaud et al., 2021; Toubiana et al., 2021), which calculated the local average log2FC of transcripts in a sliding window of 1 Mb with a 50-kb step size and applied a bootstrap method to detect regions of significant deviation (*p* < 0.05). We then counted the TE copies expressed in the testes and ovaries according to 500-kb windows and visualized the distributions of co-expression clusters and sex-biased TEs by RectChr (v1.36; https://github.com/BGI-shenzhen/RectChr).

### Identification of *dmrt* family genes for phylogenetic analysis

We applied two strategies to obtain the protein sequences of *dmrt* family genes in one mammal (*Homo sapiens*, Hs), one cartilaginous fish (*Amblyraja radiata*, Ara), 18 representative teleost fishes, including *Danio rerio* (Dre), *O. latipes* (Ola), *Gasterosteus aculeatus* (Gac), *Lepisosteus oculatus* (Loc), *Albula glossodonta* (Agl), *Anguilla japonica* (Aja), *Arapaima gigas* (Agi), *Clarias batrachus* (Cba), *Clupea harengus* (Cha), *Gambusia affinis* (Gaf), *Heterotis niloticus* (Hni), *Ictalurus punctatus* (Ipu), *Mastacembelus armatus* (Mar), *Megalops atlanticus* (Mat), *Megalops cyprinoides* (Mcy), *Oreochromis aureus* (Oau), *Scophthalmus maximus* (Sma) and arowana (Sfo) (Supplementary Table S1).

For those species with public annotations, we downloaded related gene sequences from the NCBI (Supplementary Table S2) or extracted the sequences through BLASTP (Ye et al., 2006) for using as the reference sequences. Then we employed GeneWise (Birney et al., 2004) to predict related protein-coding sequences in the arowana genome using the reference protein sequences, and obtained neighboring genes from the genome annotation or using BLAST with an E-value of 1e-5 against the arowana genome in order to validate the synteny of *dmrt* genes. We converted coding sequences (CDS) to protein sequences and used MUSCLE v3.8 (Edgar, 2004) to perform global alignments. RaxML (Stamatakis, 2006) was employed to construct a gene-family phylogenetic tree with the PROTGAMMAAUTO model. We also searched the domains of these protein sequences by using NCBI Batch CD-Search and generated visualizations via iTOL (Letunic and Bork, 2021) and IBS 2.0 (Xie et al., 2022).

## Results

### Summary of the sequencing data and quality analysis

Transcriptome sequencing (RNA-Seq) of the six libraries, constructed in triplicates from ovaries and testes, yielded 32.16 Gb and 29.76 Gb of raw reads, respectively. After data filtering, we obtained 29.43 Gb of clean reads for three ovarian samples and 27.72 Gb of clean reads for three testis samples. In addition, the average percentages of bases with quality values greater than 20 (Q20) and 30 (Q30) in the six samples accounted for 97.51% and 90.45% of the total bases, respectively. The average mapping rates of transcriptome reads were 95.91% per ovary sample and 94.86% per testis sample, independently (Table 1).

**TABLE 1:** Summary of the sequencing reads of gonad transcriptomes in Asia arowana.

Sample	Total raw reads (Mb)	Total raw bases (Gb)	Total clean reads (Mb)	Total clean bases (Gb)	Clean reads Q20(%)	Clean reads Q30(%)	Total mapping (%)
ovary2376	42.65	5.86	39.08	5.86	97.76	91.32	93.01
ovary2380	86.31	11.94	79.59	11.94	97.32	90.05	97.19
ovary2393	84.79	11.63	77.51	11.63	97.04	89.16	97.53
testis2331	76.56	10.69	71.28	10.69	97.54	90.43	94.97
testis2301	65.57	9.18	61.17	9.18	97.55	90.41	94.48
testis2312	55.82	7.85	52.32	7.85	97.82	91.31	95.13

### Identification and enrichment of DEGs in ovaries and testes

**FIGURE 1:**
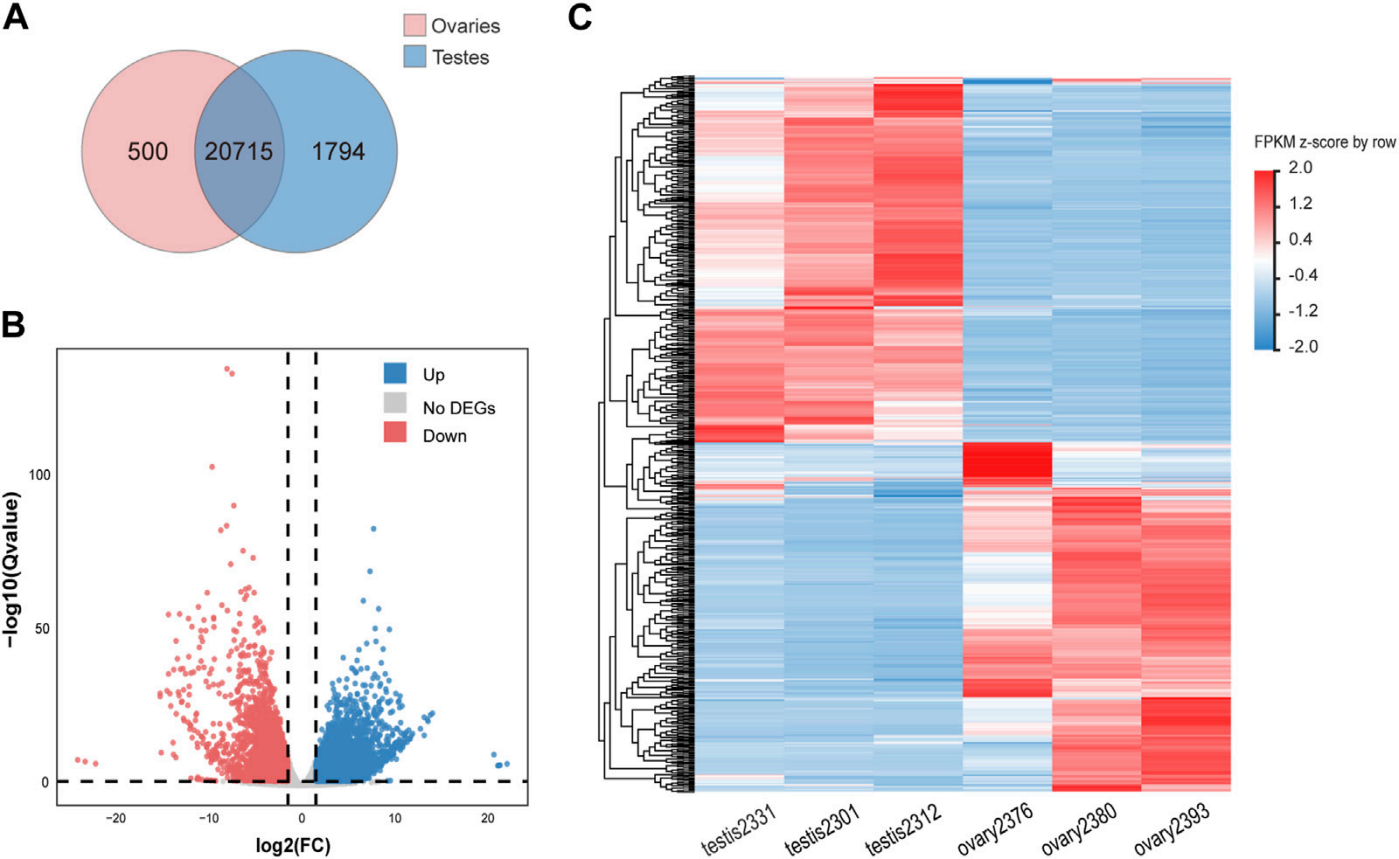
Differentially expressed genes (DEGs) obtained from the RNA-seq data of ovaries and testes. **(A)** A Venn diagram showing both expressed, testis-specific expressed, and ovarian-specific expressed genes. **(B)** A volcano plot showing number of up-/down-expressed DEGs in testes versus ovaries. Significantly upregulated (FDR < 0.05) genes in females and males are indicated by red and blue dots respectively, while genes without significant difference between sexes (FDR >0.05) are represented by gray dots. **(C)** A heatmap depicting hierarchical clustering of DEGs in gonads of Asia arowana. Here we chose the top 500 highest expressed genes in testes and ovaries, respectively; each column represents an individual, and each row represents a gene. The color scale indicates relative expression.

A total of 23,009 expressed genes were detected in our research. Among the expressed genes, a total of 20,715 genes are expressed in both males and females, while 500 and 1,794 genes are specifically expressed in males and females respectively (Figure 1A). In total, 8,872 expressed genes (38.56%) were found to be sex-biased, including 5,153 (22.40%) upregulated DEGs and 3,719 (16.16%) downregulated DEGs in testes compared with ovaries (Figures 1B, C). The remaining 14,137 genes were expressed without significant difference between genders.

**FIGURE 2:**
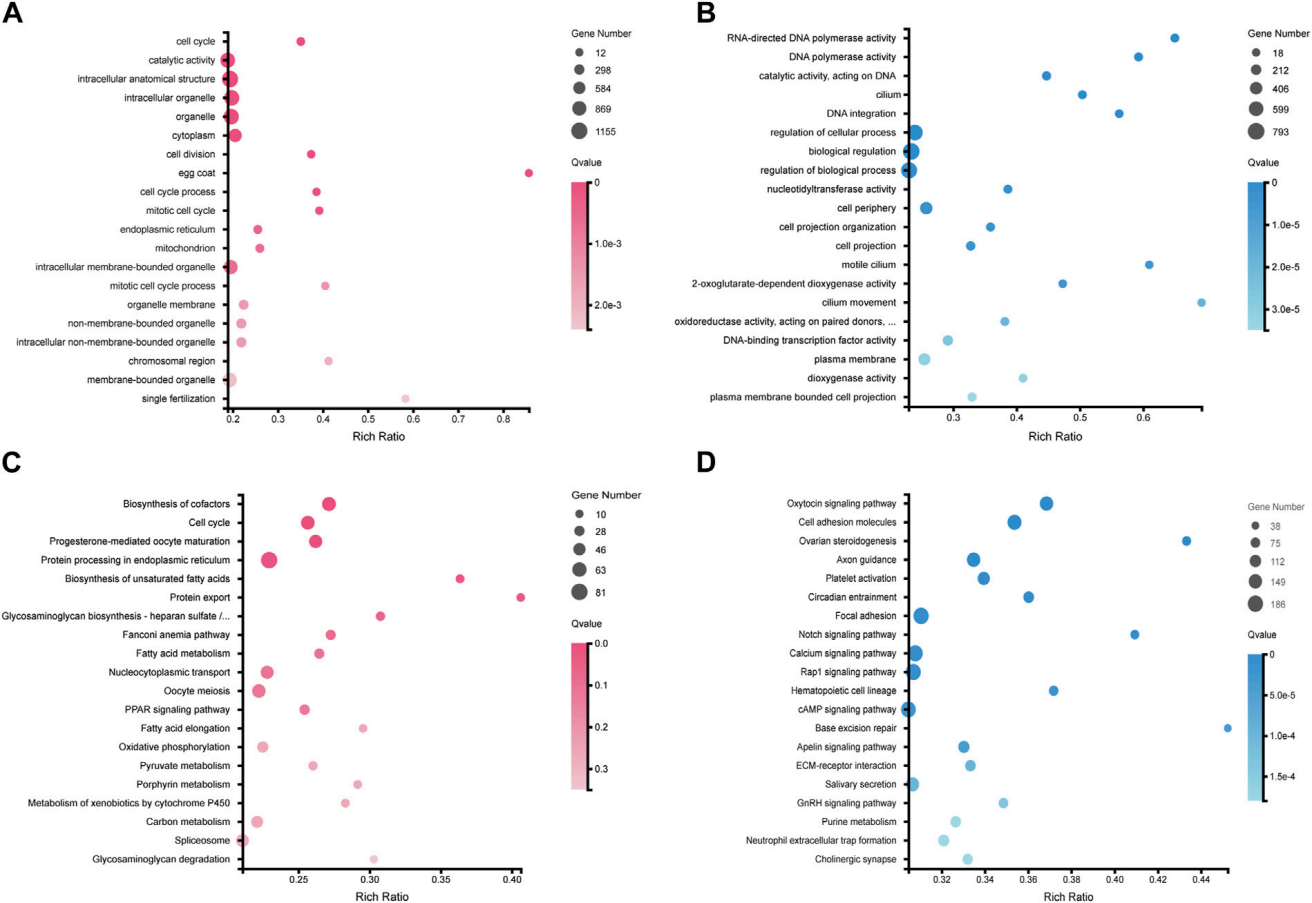
Top20 GO and KEGG pathway enrichment of DEGs in ovaries and testes. **(A)** GO enrichment of upregulated DEGs in ovaries. **(B)** GO enrichment of upregulated DEGs in testes. **(C)** KEGG pathway enrichment of upregulated DEGs in ovaries. **(D)** KEGG pathway enrichment of upregulated DEGs in testes.

GO functional annotation and KEGG pathway annotation were further performed on the DEGs. A total of 5,651 DEGs (2,612 female-biased genes and 3,039 male-biased genes) were assigned to GO terms (Supplementary Figure S1), and 3,479 DEGs were annotated to KEGG pathways (Supplementary Figure S2). GO enrichment analysis showed that ovary-biased DEGs were enriched in GO terms such as cell cycle (GO:0007049), egg coat (GO:0035805) and single fertilization (GO:0007338) (Figure 2A; Supplementary Table S3), while testis-biased DEGs were enriched in GO terms such as RNA-directed DNA polymerase activity (GO:0003964), motile cilium (GO:0031514) and regulation of cellular process (GO:0050794) (Figure 2B; Supplementary Table S4). Through KEGG pathway enrichment analysis, we observed that upregulated DEGs in ovaries were significantly enriched in cell cycle (ko04110), oocyte meiosis (ko04114), progesterone-mediated oocyte maturation pathways (ko04914) and TGF-β signaling pathway (ko04350) (Figure 2C; Supplementary Table S5), while upregulated DEGs in testes were enriched in some pathways including oxytocin signaling pathway (ko04921), focal adhesion (ko04510), cell adhesion molecules (ko04514), calcium signaling pathway (ko04020) (Figure 2D; Supplementary Table S6).

### Expression patterns of sex determination and sexual differentiation related genes

**FIGURE 3:**
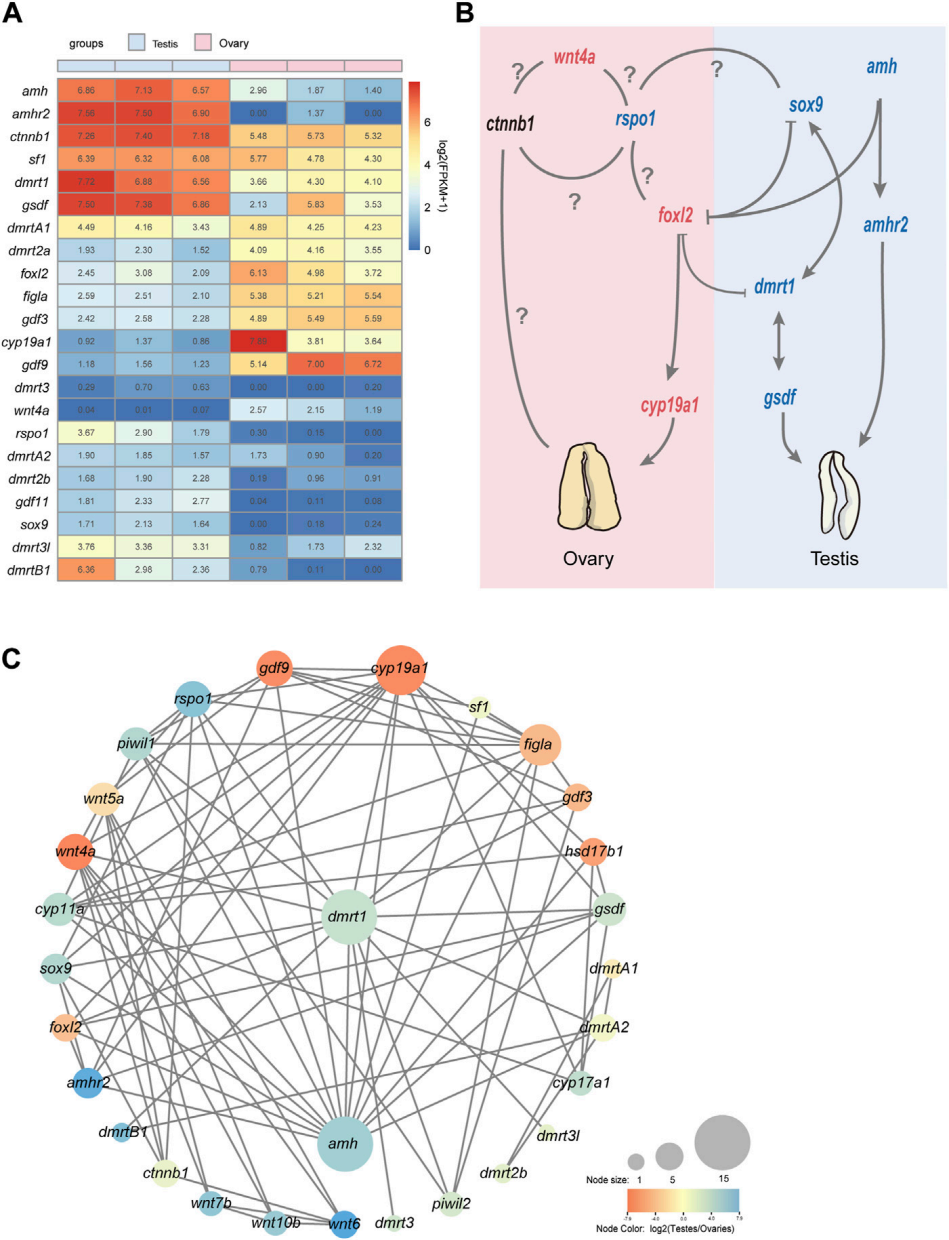
An overview of sex-related genes and a predicted regulatory network in Asia arowana. **(A)** The expression profiles of sex determination or differentiation-related genes in gonads of Asia arowana. **(B)** A predicted regulatory network for sex development in Asia arowana. Genes with upregulated expression in females and males are marked in red and blue, respectively. Note that *dmrt1* and *amh* are the key male sex regulators, while *foxl2* and *cyp19a1a* can induce female development. Symbols: ⊥: inhibition; →: stimulation; ?: unclearly. **(C)** PPI network analysis of sex determination or differentiation-related genes. Each node size reflects the number of interacting proteins, and various colors represent the differential expression profiles.

Based on the functional annotations results of NCBI NR database as well as KEGG Orthology and enrichment analyses, we performed a further comparative analysis to detect that some well-known candidate sex determination or sexual differentiation-related genes and members of the TGF-β signaling pathway showed significant sexual dimorphism in gonadal expression. Those genes related to testis differentiation as well as sperm maturation and maintenance of teleost, such as *dmrt1*, *dmrtB1, sox9*, *cyp11a* (cholesterol side-chain cleavage enzyme), *cyp17a* (steroid 17-alpha-hydroxylase/17,20 lyase-like), *amh* and its receptor *amhr2* were detected with high transcription in testes; *foxl2*, *figla* (factor in the germline alpha), *cyp19a1* (aromatase), and *gdf9*, related to ovary differentiation, were significantly expressed in ovaries compared with testes (Figure 3A). Several members of *wnt* family also showed sex-biased in gonads. For example, *wnt4a* and *wnt11* showed a female bias, but *wnt5b*, *wnt6*, *wnt7b*, *wnt8b*, and *wnt10b* upregulated in testes; however, *wnt4b* was expressed without differential difference in these samples (Table 2; Figure 3A).

**TABLE 2:** Summary of sex determination and sex differentiation associated genes in Asia arowana.

Gene name	Gene description	log2(Testes/Ovaries)	FDR	Sex-bias
*hsd17b7*	3-keto-steroid reductase/17-beta-hydroxysteroid dehydrogenase 7	1.64	4.66E-03	Male
*piwil2*	piwi-like protein 2	1.81	1.92E-04	Male
*gsdf*	growth differentiation factor 6	2.23	2.24E-03	Male
*dmrt1*	doublesex- and mab-3-related transcription factor 1	2.50	1.41E-09	Male
*wnt5b*	wingless-type MMTV integration site family, member 5b	2.76	3.25E-02	Male
*cyp17a1*	steroid 17-alpha-hydroxylase/17,20 lyase	2.77	6.04E-04	Male
*cyp11a*	cholesterol side-chain cleavage enzyme	3.14	2.97E-05	Male
*hsd17b3*	testosterone 17-beta-dehydrogenase 3	3.42	2.15E-02	Male
*piwil1*	piwi-like protein 1	3.46	1.01E-09	Male
*sox9*	transcription factor SOX-9a	3.46	5.38E-04	Male
*sox2*	transcription factor SOX-2	3.82	7.20E-02	Male
*amh*	muellerian-inhibiting factor	4.21	3.31E-17	Male
*wnt10b*	wingless-type MMTV integration site family, member 10b	4.64	7.22E-02	Male
*wnt2b*	wingless-type MMTV integration site family, member 2b	4.65	4.21E-06	Male
*wnt8b*	wingless-type MMTV integration site family, member 8b	4.87	3.95E-02	Male
*wnt7b*	wingless-type MMTV integration site family, member 7b	5.01	1.62E-02	Male
*rspo1*	R-spondin-1	5.41	6.82E-10	Male
*dmrtB1*	doublesex- and mab-3-related transcription factor 6	5.72	1.31E-04	Male
*gdf11*	growth/differentiation factor 11	5.90	8.65E-11	Male
*amhr2*	anti-Muellerian hormone type-2 receptor	7.42	9.60E-04	Male
*wnt6*	wingless-type MMTV integration site family, member 6	7.95	1.33E-08	Male
*wnt4a*	wingless-type MMTV integration site family, member 4	−7.26	8.33E-17	Female
*cyp19a1*	aromatase	−6.97	2.93E-11	Female
*gdf9*	growth/differentiation factor 9	−6.82	1.48E-24	Female
*hsd17b1*	estradiol 17-beta-dehydrogenase 1	−5.61	1.58E-07	Female
*wnt11*	wingless-type MMTV integration site family, member 11	−5.46	1.00E-24	Female
*hsd17b12*	17beta-estradiol 17-dehydrogenase/very-long-chain 3-oxoacyl-CoA reductase	−5.09	9.02E-24	Female
*sox32*	transcription factor SOX32	−4.76	2.62E-02	Female
*figla*	factor in the germline alpha isoform	−4.16	2.38E-23	Female
*gdf3*	growth/differentiation factor 3	−4.13	2.81E-18	Female
*foxl2*	forkhead box protein L2	−3.67	3.24E-08	Female
*dmrt2a*	doublesex- and mab-3-related transcription factor 2a	−3.39	3.90E-23	Female
*wnt5a*	wingless-type MMTV integration site family, member 5a	−1.80	7.97E-03	Female
*hsd17b4*	peroxisomal multifunctional enzyme type 2	−1.79	7.72E-04	Female
*hsd17b10*	3-hydroxyacyl-CoA dehydrogenase type-2	−1.63	3.75E-03	Female
*gdf7*	growth/differentiation factor 7	−1.65	4.54E-01	-
*hsd17b8*	estradiol 17-beta-dehydrogenase 8	−0.98	8.73E-02	-
*dmrtA1*	doublesex- and mab-3-related transcription factor A1	−0.91	1.17E-02	-
*foxl3*	forkhead box protein L3	−0.87	8.19E-01	-
*sox17*	transcription factor Sox-17	−0.82	4.37E-01	-
*wnt9a*	wingless-type MMTV integration site family, member 9a	−0.79	8.39E-01	-
*wnt16*	wingless-type MMTV integration site family, member 16	−0.59	6.29E-01	-
*sox11*	transcription factor SOX-11	0.08	9.49E-01	-
*sox5*	transcription factor SOX-5	0.43	6.09E-01	-
*sox7*	transcription factor Sox-7	0.43	8.24E-01	-
*dmrtA2*	doublesex- and mab-3-related transcription factor A2	0.43	7.21E-01	-
*sox6*	transcription factor SOX-6	0.48	5.19E-01	-
*sf1*	splicing factor 1	0.54	2.92E-01	-
*ctnnb1*	catenin beta-1	0.83	1.06E-03	-
*dmrt3*	doublesex- and mab-3-related transcription factor 3	2.02	3.89E-01	-
*dmrt3l*	doublesex- and mab-3-related transcription factor 3a-like	1.06	2.03E-01	-
*sox8*	transcription factor Sox-8	1.06	8.33E-01	-
*sox21*	transcription factor SOX-21	1.11	1.59E-01	-
*dmrt2b*	doublesex- and mab-3-related transcription factor 2b	1.16	2.53E-01	-
*gdf5*	growth/differentiation factor 5	1.34	4.51E-01	-
*sox13*	transcription factor SOX-13	1.39	1.42E-01	-
*wnt7a*	wingless-type MMTV integration site family, member 7a	1.84	4.21E-01	-
*sox3*	transcription factor Sox-3	3.20	3.49E-01	-

The STRING database was utilized to construct a PPI network for potential sex-related genes among the DEGs. It was revealed that 31 potential sex-related genes exhibited intricate interaction relationships. Notably, *dmrt1* and *amh* were associated with 15 sex-related genes, *cyp19a1* collaborated with 13 genes, and Foxl2 interacted with five genes. *Dmrt1* not only interacted closely with testis-upregulated genes (such as *amh*, *sox9*, *dmrtB1*, and *rspo1*), but also with female sex-related genes (like *wnt4a*, *foxl2*, *figla*, and *cyp19a1*). Furthermore, *foxl2* interacted with its downstream functional gene *cyp19a1*, as well as with *dmrt1*, *amh*, and *amhr2* (Figures 3B, C).

### Identification of TE copies and families with sex-biased expression

**FIGURE 4:**
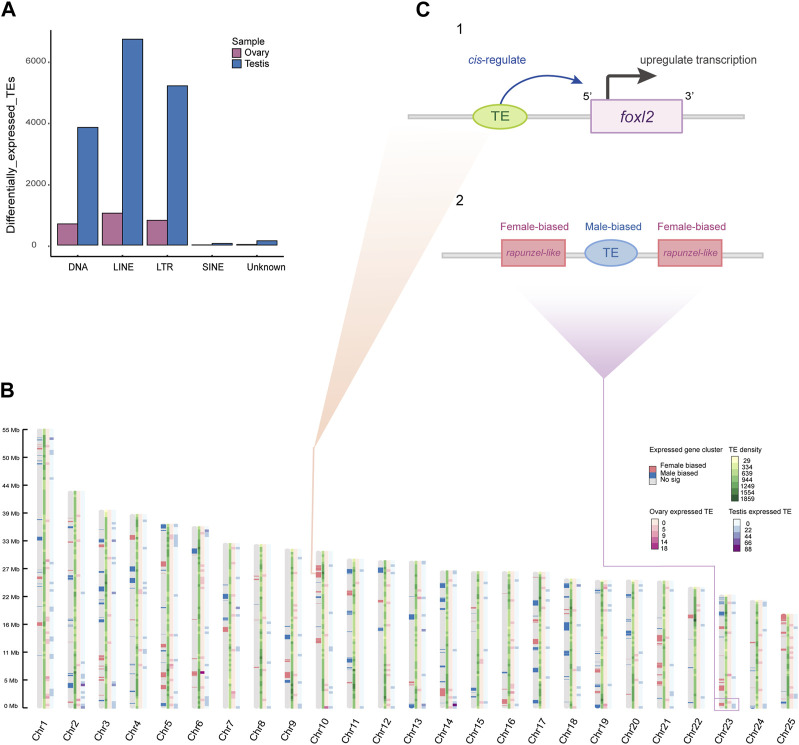
Sex-biased expressed TEs and gene expression clusters. **(A)** A bar plot of differentially expressed TEs in ovaries and testes (categorized by TE family). **(B)** Genome-wide sex-biased gene clusters and TEs density across sliding windows. The color pattern on each chromosome represents gene expression cluster, TE density, ovary-biased expressed TE density, and testis-biased expressed TE density, respectively. **(C)** The predicted regulative pattern of expressed TE at upstream of the *foxl2* (1). The testis-biased TE located in neighborhood of ovary-biased expressed genes (2).

We characterized gonadal expression of TEs in order to analyze TE expression relative to gene expression. Unlike protein-coding genes that have an approximately equal proportions of expression in both sexes, TEs appeared to have a significant testicular bias. In brief, 18,490 TE copies from the transcriptomes were detected with significantly differential expression in the gonads, of which 15,930 (86.15%) were testis-biased, while 2,560 (13.84%) were ovarian biased. We classified these testis- or ovary-biased TEs into five subfamilies by TE annotation subsequently (Figure 4A). Interestingly, we observed that sex-biased expressed genes often clustered in a genome-wide manner. And in some chromosomes, TE copies located near ovary-biased gene regions are still showing testis expression (such as the testis-biased TE is located between the two copies of the ovary-biased *rapunzel-like* on Chr23; Figure 4B, C-2), which is consistent with the trend that the overall expression of TEs in the genome is more testis-biased (Figure 4A). Nevertheless, we also found some TE copies expressed near female related genes, such as an expressed *LTR retrotransposon* and an expressed DNA transposon at the upstream of the *foxl2* (Figure 4C-1).

### Summary of *dmrt* family genes and related phylogenetic tree

**FIGURE 5:**
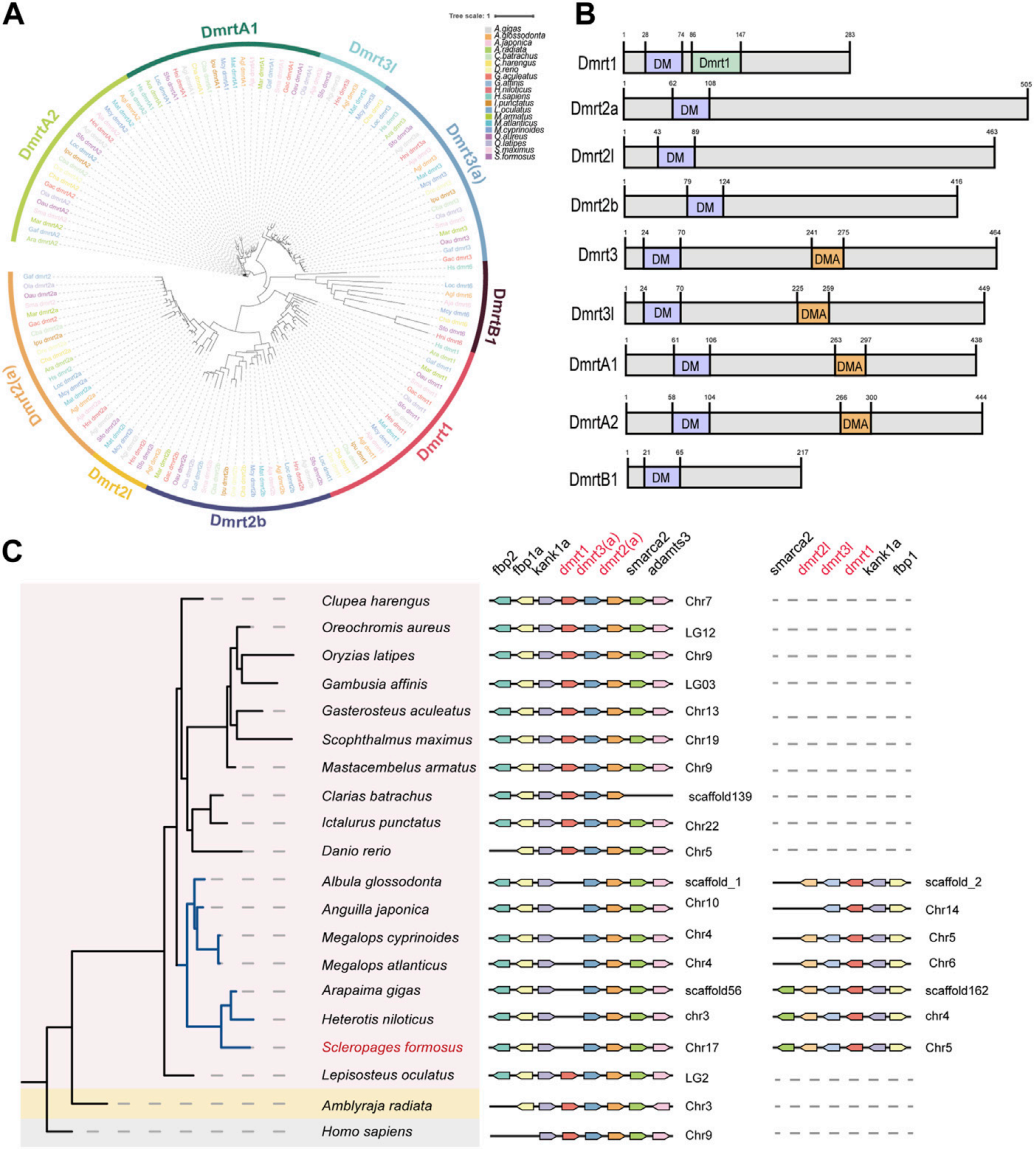
The phylogeny of vertebrate *dmrt* gene family and a synteny analysis of the conserved *dmrt1*-*dmrt3*-*dmrt2* gene clusters. **(A)** A phylogenetic tree of the *dmrt* gene family in 20 representative species (constructed by the maximum likelihood method). **(B)** A schematic diagram of conserved domain structures in various DMRT proteins. **(C)** The synteny of *dmrt1*-*dmrt3*-*dmrt2* gene clusters.

Since multiple copies of *dmrt* family genes were detected by the transcriptome sequencing, we scanned the *dmrt* gene family in many representative genomes of arowana and some other bony fishes. A total of nine members from the *dmrt* gene family were identified in arowana (Figure 5A) and named based on the nomenclature of teleost *dmrt* genes. All of these identified DMRT proteins contain a conserved DM (Doublesex and Mab-3) domain (near the N-terminus in Figure 5B). Meanwhile, Dmrt1 has an additional DMRT1 domain, as well as Dmrt3, Dmrt3L, DmrtA1 and DmrtA2 own a special DAM domain (middle in Figure 5B). Furthermore, we observed a somehow conserved *dmrt1*-*dmrt3*-*dmrt2* cluster in the examined 20 species (see the middle panel in Figure 5C), while those species of Osteoglossus and Elopomorpha have another *dmrt2*-*dmrt3*-*dmrt1* gene cluster (see the left panel in Figure 5C). Based on the neighboring genes of both gene clusters, we infer that the gene clusters of these two groups are *dmrt1*-*dmrt3l*-*dmrt2l* and *dmrt3*-*dmrt2a*, respectively (Figure 5C).

The expression patterns of *dmrt* genes were investigated among testes and ovaries. Obviously, the transcription levels of *dmrt1* and *dmrtB1* in the testes were significantly higher than those in ovaries, *dmrt3l* was upregulated in the testes, while *dmrt2l* was not detectable in transcription (Figure 3A). Surprisingly, in the *dmrt3*-*dmrt2a* gene cluster (middle panel of Figure 5C), the *dmrt3* is slightly expressed in the testis but undetectable in the ovary, while *dmrt2a* is highly transcribed in the ovary (Figure 3A).

## Discussion

Transcriptomics is an effective method to obtain gene regulatory networks among different individuals. In this study, we employed the RNA-Seq technology for transcriptomics analysis, and many genes related to gonad development were identified and characterized to improve our understanding of their sex-related functions from a molecular perspective. Generally speaking, the fish sex determination mechanism usually involves complex biological processes, including a series of genes that promote or maintain the development of gonads into testes or ovaries. The sexual dimorphism in expression identified in this study, especially for those previously reported candidate genes that are related to sex determination and sexual differentiation (Kitano et al., 2023), may be effective indicators for sex prediction in Asian arowana.

The Wnt signaling is a crucial pathway to regulate cell proliferation, differentiation, embryonic development, and folliculogenesis. Recent genetic research has identified the *wnt4* as a vital regulator of the Wnt/β-catenin signaling pathway during sex determination (Kossack et al., 2018; Farhadi et al., 2021). The most important member *wnt4* gene has been identified in zebrafish (Kossack et al., 2018), medaka (LI et al., 2012) and various other teleost fishes. Due to the teleost-specific whole-genome duplication (WGD) events, there are two paralogs in most teleost, *wnt4a* and *wnt4b*, both of which play essential roles in the development of female gonads, ovarian growth, early male development, and sex transition (Hu et al., 2014). We found that several *wnt* genes showed sex-biased expression patterns in arowana. For example, the expression of *wnt4a* (log2FC = −7.26) and *wnt11* (log2FC = −5.46) that are related to ovary differentiation were upregulated in ovaries; the expression of *wnt6* (log2FC = 7.95), which could initiate Wnt/β-catenin signaling to control the proliferation of undifferentiated spermatogonia in mouse (Takase and Nusse, 2016), was also testis-biased. *rspo1* showed a sexually dimorphic expression pattern with significantly higher expression in testes over ovaries in our present study (log2FC = 5.41). It is a potential female-determining gene in mammals that can regulate the important Wnt/β-catenin signaling pathway; loss of *rspo1* can lead to female sex reversal. However, the expression pattern of *rspo1* in fish seems not very conservative. For instance, in many teleost fishes such as zebrafish, medaka, and half-smooth tongue, *rspo1* shows a female bias, while in some ancient fishes (like *Acipenser stephensi*, coelacanth, and lungfish) and some sex-changed teleost fishes (like *Notolabrus celidotus*) shows a male bias(Biscotti et al., 2018; Zhang et al., 2020; Muncaster et al., 2023), implying that *rspo1* may have other gender-related functions in various fishes.

The TGF-β signaling pathway is a large family comprising many members responsible for regulation of various processes including tissue and organ formation, reproductive development, cell growth, proliferation, and differentiation (Pan et al., 2021). In fish, this critical pathway often plays a key role in regulating important physiological processes, such as embryonic development, tissue regeneration, immune regulation, chondrogenesis, as well as sex determination and sexual differentiation (Pan et al., 2021; Kitano et al., 2023). Some members belonged to the TGF-β superfamily, such as *amh*, *amhr2*, and *gsdf* were highly expressed in males (Figure 3A), which indicates that these pathways should play a significant role in sexual differentiation of Asian arowana. During ovarian development, the interaction among granulosa cells, theca cells, and oocytes is crucial for follicular development, steroidogenesis, and female oogenesis (Richards, 2018). The forkhead-box L2 (*foxl2*) transcription factor is one of the earliest known markers of granulosa cell differentiation (Cocquet et al., 2002), and it has been determined to be a major gene of sex determination and maintenance in various teleost (Cui et al., 2017; Zhang et al., 2017; Yuan et al., 2021). In addition, the *foxl2* can directly bind to the promoter region of the aromatase gene *cyp19a1a* to promote its expression, or indirectly regulated expression of *cyp19a1a* through interaction with *sf1* (splicing factor 1), resulting in estrogen (E2) production to promote ovarian differentiation (Wang et al., 2007; Zhang et al., 2017; Yuan et al., 2021). In our present study, *folx2* (log2FC = −3.67) and *cyp19a1* (log2FC = −6.97) were found to be highly upregulated in the ovary compared with the testis (Figure 3A), showing a similar expression pattern to other fish species (Zhang et al., 2019; Mustapha et al., 2022). Through a PPI analysis, we found that most of the potential sex-related genes among the DEGs exhibit interaction relationships, suggesting that these genes may play functional roles in sex differentiation and development, potentially being directly or indirectly involved in gonadal development or differentiation. Notably, *dmrt1*, *amh*, as well as *cyp19a1* (and its upstream regulator *foxl2*) interact with multiple detected potential sex-related genes, indicating that these gene play central regulatory roles in sex differentiation in Asia arowana. In mammals, *Dmrt1* directly represses *Foxl2* expression in the testis, and *Foxl2* is required for repressing *Dmrt1* expression in the ovary (Matson et al., 2011). Previous studies on tilapia and zebrafish found that *dmrt1* has an antagonistic effect on the expression of *foxl2* and *cyp19a1* (Wang et al., 2010). *Dmrt1* can directly repress the expression of *foxl2* and *cyp19a1* in somatic cells of tilapia, while in females *foxl2* activates the expression of *cyp19a1* and inhibits expression of *dmrt1*. In addition, studies have found that *foxl2* has ovary-enriched expression in zebrafish, just like in mammals, and *dmrt1* is required for its downregulation. Therefore, it is speculated that the antagonistic relationship between *foxl2* and *dmrt1* in the sex determination process may be conserved among vertebrates (Webster et al., 2017). We have observed that *dmrt1* not only interacts with male-biased gene, such as *amh* and *sox9*, but also engages with *cyp19a1* and *foxl2*, suggesting that the antagonism may also exist in Asian arowana. However, due to limited research on Asian arowana, some protein-protein interactions may require further experimental validation (like Dmrt2a). According to the sex-biased DEGs detected in our present study and the sex relevant pathways in amphioxus and vertebrates (Pan et al., 2021; Curzon et al., 2023; Huang et al., 2023), we infer that *wnt4a*, *foxl2* and *cyp19a1* may play the major role in promoting ovarian differentiation of Asia arowana, while the expression and regulation of *sox9*, *dmrt1*, *gsdf*, *amh* and *amhr2* possibly play an important role in development and differentiation of testis. Based on this inference, a potential regulatory network of Asian arowana sexual development was proposed (Figure 3B).

It is well known that TEs could regulate gene expression (Feschotte, 2008; Sundaram and Wysocka, 2020; Gebrie, 2023), and previous studies reported that the closer TE copy to the target gene, the higher correlation of their expressions (Dechaud et al., 2021). In mammalian genomes, TEs are an important source of various cis-regulatory sequences; 20% of the cis-regulatory elements (CREs) in the human genome may have been taken from TEs (Sundaram and Wysocka, 2020), and TEs often contribute to zebrafish cis-regulatory elements, tissue-specific expression and alternative promoters (Lee et al., 2022). Researchers have found that the fourth exon (Ex4) of the sex-determining gene *dm-W* in the African clawed frog (*Xenopus laevis*) originated from a non-coding fragment of the *hAT-10* family of DNA transposons (Hayashi et al., 2022). An experimental evidence from sablefish (*Anoplopoma fimbria*) demonstrated that a TE insertion in the promoter region of *gsdfY* produced allelic diversification by bringing a cis-regulatory module, leading to transcriptional reprofiling and generating a new sex-determined gene for this species (Herpin et al., 2021). The regulatory elements of many genes contain TE sequences, which are involved in the regulation of gene expression. Some studies have reported that TEs can be expressed in multiple tissues (Brunet et al., 2018; Chang et al., 2022; Xu et al., 2023). However, because they align to multiple genomic loci, many RNA-seq reads derived from TEs are often discarded before data analysis. To resolve this problem, several computational tools have been developed (Jin et al., 2015; Jeong et al., 2017; Yang et al., 2019; Lanciano and Cristofari, 2020). We therefore observed a DNA transposon and an LTR transposon located at the promoter region of *foxl2* expressed in the arowana ovary (Figure 4C), suggesting co-regulation of the cis-regulatory elements to enhance the neighboring sex-biased gene(s) and/or sex-biased TEs themselves on these two types of sequences, as well as the *foxl2* may play a significant functional role in ovarian differentiation of arowana.

*Dmrt1*, an important member of the *dmrt* gene family, is essential for maintaining male-specific germ cells and testis differentiation (Matson and Zarkower, 2012; Cui et al., 2017). It not only participates in sex regulation in mammals, but also acts as a ubiquitous conserved sex regulation factor in other non-mammalian vertebrates including fishes. A recently published paper reported no orthologous gene of *dmrt1* in amphioxus (Huang et al., 2023), while previous studies on the *dmrt* family of diverse animals found that *dmrt1* and *dmrtB1* are vertebrate-specific genes, and they may have arisen in vertebrates through WGD events (Mawaribuchi et al., 2019). Various studies on non-mammalian vertebrates have proved that *dmrt1* or its paralogous genes control gonadal sex determination and differentiation with different mechanisms (Smith et al., 2009; Yoshimoto et al., 2010; Cui et al., 2017; Mawaribuchi et al., 2019). From the TF annotation of arowana transcriptomes, we identified multiple copies of *dmrt*, such as *dmrt3* and *dmrt2a*. Through gene annotation and genome scanning of one elasmobranch and 18 bony fishes, we observed the conserved *dmrt1*-*dmrt3*-*dmrt2* gene cluster among various vertebrates, and some species of the order Osteoglossus and Elopomorpha also have another *dmrt3*-*dmrt2* gene cluster (Figure 5C). Previous studies have shown that *dmrt1* may have emerged after a WGD event in vertebrates, and based on our findings we speculate that the *dmrt* gene clusters in these two basal teleost species are likely to come from the teleost-specific WGD event (3R-WGD).

It seems that the expanded *dmrt* gene cluster remains in this clade for continuous function after the 3R-WGD. This confirms that the two groups are sister branch to each other, and this branch forms as sister groups to all the other teleost (Parey et al., 2023). The gene cluster identified in the Asian arowana was named as *dmrt1*-*dmrt3l*-*dmrt2l*, based on the names of related genes published in the NCBI, and another cluster is named as *dmrt3*-*dmrt2a* (Figure 5C). Interestingly, *dmrt1*-*dmrt3l* in the *dmrt1*-*dmrt3l*-*dmrt2l* gene cluster was upregulated in the arowana testis (log2FC = 2.50, log2FC = 1.06), while *dmrt2l* was undetectable in both testes and ovaries; in the *dmrt3*-*dmrt2a* gene cluster, the *dmrt3* gene is slightly expressed in the testes but without expression in the ovary, while *dmrt2a* is highly expressed in the ovary (log2FC = −3.39) (Figure 3A). These expression trends are very similar to those in other teleost fishes, indicating that *dmrt1* and *dmrt2* located in different clusters may be critical players in the sexual differentiation of Asian arowana: one of the gene clusters may be associated with male development, while another is potentially associated with female development.

## Conclusion

Based on gonadal transcriptome sequencing and transcriptomic comparisons, we characterized a set of differentially expressed genes and pathways for potential involvement in sex determination or differentiation in Asia arowana. This sexual dimorphism is valuable for ascertaining the differential expression patterns of sex-related genes and enriching the genetic resources of this ornamental fish species. Our transcriptome data on sex-related genes can also promote the exploration of molecular mechanisms of gonadal development and sex determination in Asian arowana, which provides valuable references for practical gender identification and one-to-one breeding programs so as to expand fish number for a rapid elevation of worldwide economic value.

## Data Availability

The datasets presented in this study can be found in online repositories. The names of the repository/repositories and accession number(s) can be found below: NCBI under accession numbers: SRR20631749-SRR20631754.
