# Supplementary material for: Gonadal transcriptome sequencing reveals sexual dimorphism in expression profiling of sex-related genes in Asian arowana (Scleropages formosus)
Source: Front Genet. 2024 Apr 11;15:1381832. doi: 10.3389/fgene.2024.1381832 (PMC11043485; doi:10.3389/fgene.2024.1381832)
Supplement: Supplementary file 3 [file Table1.DOCX]

**Supplementary Materials**


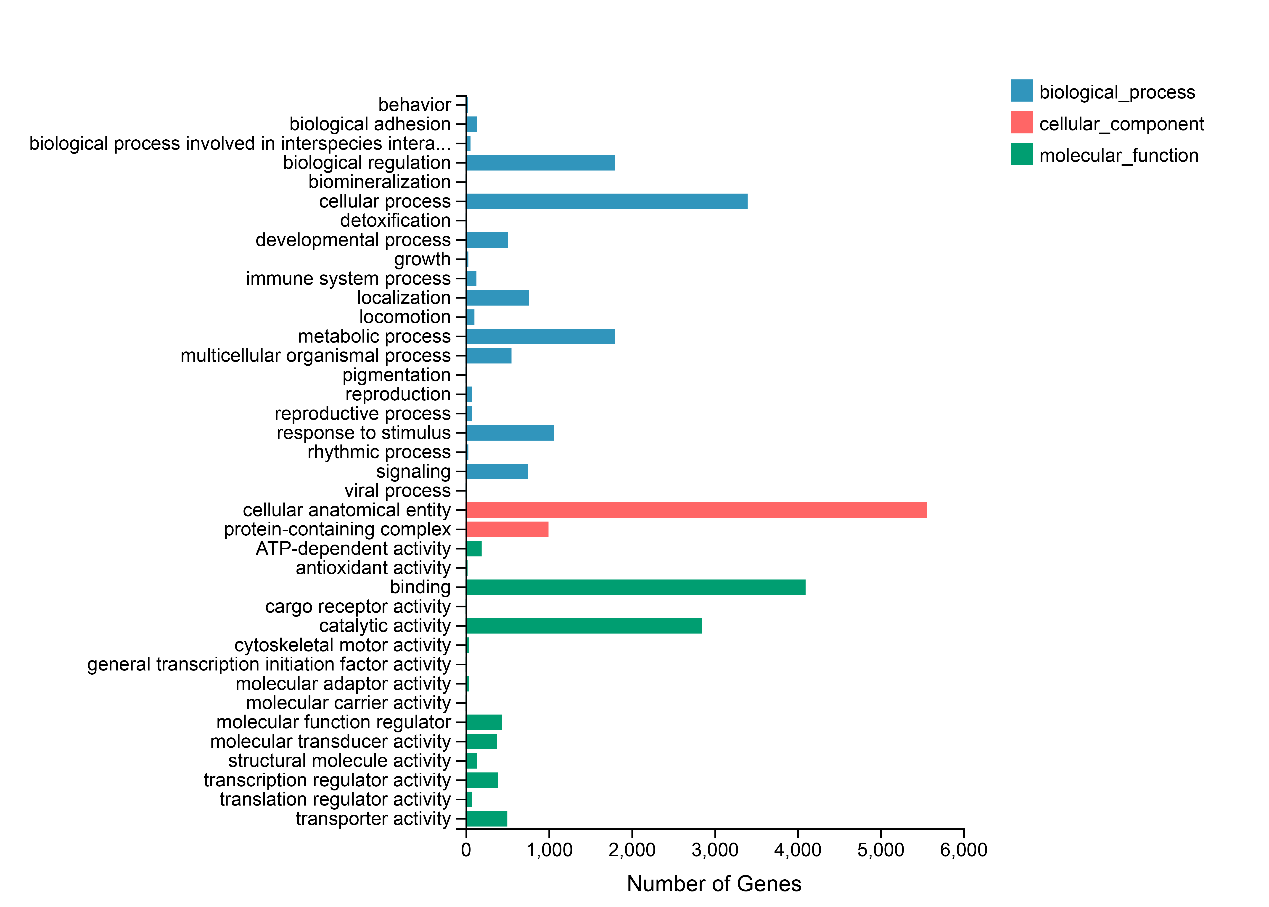


**Figure S1** GO functional annotation of DEGs.


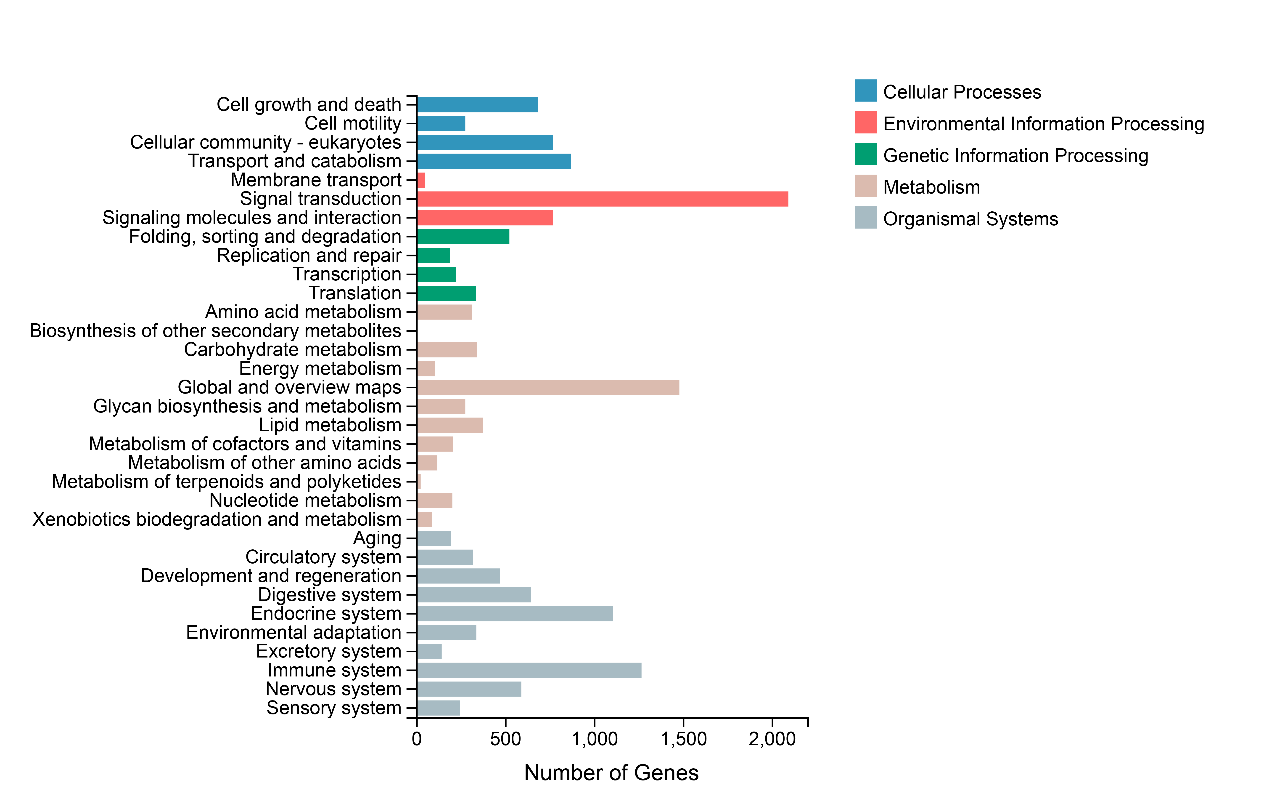


**Figure S2** KEGG pathway annotation of DEGs.

**Table S1** Genomes used for detection of the *dmrt* family.

| **Species** | **NCBI accession no.** | **Abbreviation** |
| --- | --- | --- |
| *Albula glossodonta* | GCA_019788955 | Agl |
| *Amblyraja radiata* | GCF_010909765 | Ara |
| *Anguilla japonica* | GCA_025169545 | Aja |
| *Arapaima gigas* | GCA_900497675 | Agi |
| *Clarias batrachus* | GCA_003987875 | Cba |
| *Clupea harengus* | GCF_900700415 | Cha |
| *Gambusia affinis* | GCF_019740435 | Gaf |
| *Heterotis niloticus* | GCA_018136845 | Hni |
| *Ictalurus punctatus* | GCF_001660625 | Ipu |
| *Mastacembelus armatus* | GCF_900324485 | Mar |
| *Megalops atlanticus* | GCA_019176425 | Mat |
| *Megalops cyprinoides* | GCF_013368585 | Mcy |
| *Oreochromis aureus* | GCF_013358895 | Oau |
| *Scophthalmus maximus* | GCF_022379125 | Sma |

**Table S2** Protein sequences encoded by the reference *dmrt* and its neighbor genes.

| **Species** | **Species Abbreviation** | **Gene** | **Accession No.** |
| --- | --- | --- | --- |
| *Danio rerio* | Dre | Dre*_dmrt1* | NP_991191.2 |
|  |  | Dre_*dmrt2b* | NP_001073445.1 |
|  |  | Dre_*dmrt2a* | NP_571027.1 |
|  |  | Dre*_dmrt3* | NP_001005779.2 |
|  |  | Dre*_dmrtA2* | NP_001007065.2 |
| *Gasterosteus aculeatus* | Gac | Gac_*dmrt1* | NP_001254571.1 |
|  |  | Gac_*dmrt2b* | XP_040041201.1 |
|  |  | Gac*_dmrt2* | XP_040052663.1 |
|  |  | Gac_*dmrt3* | XP_040050563.1 |
|  |  | Gac_*dmrtA1* | XP_040037725.1 |
|  |  | Gac_*dmrtA2* | XP_040041120.1 |
| *Ictalurus punctatus* | Ipu | Ipu_*dmrt6* | XP_017328630.1 |
| *Lepisosteus oculatus* | Loc | Loc*_dmrt1* | XP_015194160.1 |
|  |  | Loc_*dmrt2a* | XP_006627187.1 |
|  |  | Loc_*dmrt2b* | XP_006634975.1 |
|  |  | Loc_*dmrt3* | XP_006627186.1 |
|  |  | Loc_*dmrtA1* | XP_006630066.1 |
|  |  | Loc_*dmrtA2* | XP_015211440.1 |
|  |  | Loc_*adamts3* | XP_015195658.1 |
|  |  | Loc_*fbp1* | XP_006627184.1 |
|  |  | Loc_*fbp2* | XP_006627183.1 |
|  |  | Loc_*kank1a* | XP_006627253.2 |
|  |  | Loc_*smarca2* | XP_015195454.1 |
| *Oryzias latipes* | Ola | Ola*_dmrt1* | NP_001098150.2 |
|  |  | Ola_*dmrt2a* | XP_023813898.1 |
|  |  | Ola_*dmrt2b* | XP_004068077.2 |
|  |  | Ola_*dmrt3* | XP_023813900.1 |
|  |  | Ola_*dmrtA1* | XP_004079711.1 |
|  |  | Ola_*dmrtA2* | XP_023810346.1 |
|  |  | Ola_*adamts3* | XP_023813887.1 |
|  |  | Ola*_fbp1a* | XP_004086497.1 |
|  |  | Ola_*fbp2* | XP_004086496.1 |
|  |  | Ola_*kank1a* | XP_011471394.1 |
|  |  | Ola_*smarca2* | XP_023813897.1 |
| *Homo sapiens* | Hs | Hs*_dmrt1* | NP_068770.2 |
|  |  | Hs_*dmrt2* | NP_870987.2 |
|  |  | Hs_*dmrt3* | NP_067063.1 |
|  |  | Hs_*dmrtA1* | NP_071443.2 |
|  |  | Hs_*dmrtA2* | NP_115486.1 |
|  |  | Hs_*dmrtB1* | NP_149056.1 |
|  |  | Hs_*kank1* | NP_055973.2 |
|  |  | Hs**_***smarca2* | NP_003061.3 |

**Table S3** GO enrichment of ovary transcripts.

See a separate file.

**Table S4** GO enrichment of testis transcripts.

See a separate file.

**Table S5** KEGG pathway enrichment of ovary transcripts.

See a separate file.

**Table S6** KEGG pathway enrichment of testis transcripts.

See a separate file.
